# Comparative analysis of drop mass variance for manual and automatic delivery of large–scale eye drop poultry vaccine

**DOI:** 10.1016/j.psj.2026.107159

**Published:** 2026-05-21

**Authors:** Michael Bakker, Barbora Vraníková, Erik Jurjen Duintjer Tebbens, Zdenka Šklubalová

**Affiliations:** a Department of Biophysics and Physical Chemistry, Faculty of Pharmacy in Hradec Králové, Charles University, Akademika Heyrovského 1203, 500 05 Hradec Králové, Czech Republic; b Department of Pharmaceutical Technology, Faculty of Pharmacy in Hradec Králové, Charles University, Akademika Heyrovského 1203, 500 05 Hradec Králové, Czech Republic

**Keywords:** Eye drop, Vaccine, Poultry, Drop mass, Dropper apparatus

## Abstract

The delivery of ocular vaccines in the poultry industry is a crucial challenge, where manual eye-drop administration is prone to user-dependent variation. This leads to significant vaccine waste, increased labor costs, and potential health risks to animals. Employing robust statistical modeling, we aimed to compare a novel automated dropper apparatus against conventional methods under various influencing factors. Through multi-linear regression, we identified dispensing angle and inconsistent manual pressure as primary sources of drop mass variability in conventional systems. For flat-tip droppers, decreasing the dispensing angle led to smaller drops, while for round-tips, a tilted position (45°) altered drop mass significantly due to lateral wetting. We also demonstrated that remaining volume of the bottle could not explain the variability in drop mass, evidenced by near-zero R-squared values in regression models regardless of preparation or dispensing rate. The automated dropper exhibited superior performance, reducing drop mass standard deviation by 1.32 to 1.23 mg compared to manual methods ensuring both consistent dosing and minimizing waste. These lower standard deviations were particularly noticeable when compared to alternative dispensing techniques. These findings underscore the potential of automated systems to enhance accuracy and consistency in large-scale eye-drop poultry vaccine delivery.

## Introduction

The prevention of infectious diseases in poultry plays a crucial economic role in large-scale farming. The diseases are primarily controlled by vaccination either in a preventive manner or in the case of an outbreak (Guy and Garcia, 2008; Menendez et al., 2014). Even though the generally used intramuscular injection vaccines are highly efficient, they produce pain and require professionals for their administration. Contrary, the efficacy of oral vaccination by drinking water is less satisfactory, as vaccine degradation often occurs in the gastrointestinal tract. Wherever the entering pathogens cause respiratory tract infections, the nasal and ocular route of administration become an important vaccination strategy (Devlin et al., 2008; Tong et al., 2020; Gowthaman et al., 2020), inducing even better mucosal immunity than the parenteral one, providing the first line of defense against viral infections (Atmar et al., 2007; Tseng et al., 2009; Worrall and Sudarisman Priadi, 2009; Thilakarathne et al., 2019). Examples of such infections include avian influenza viruses (de Geus et al., 2012; Tong et al., 2001) and infectious laryngotracheitis virus (Tong et al., 2001; Menendez et al., 2014; Gowthaman et al., 2020; Mo and Mo, 2025)

Infectious laryngotracheitis (ILT) represents an upper respiratory, world-globally distributed, highly contagious viral respiratory tract disease of poultry caused by a type I Gallid alphaherpesvirus (Tong et al., 2001; Menendez et al., 2014; García, 2017; Gowthaman et al., 2020; Mo and Mo, 2025). Although there is no known risk of human infection by ILT, the disease is characterized in poultry by signs of respiratory depression, gasping, expectoration of bloody mucus, and a high level of mortality associated with high economic losses (Bagust and Guy, 1997; Guy and Garcia, 2008; Gowthaman et al., 2020). Administration of the vaccine by drinking water or spraying to control ILT are the often-used ways in pullet farming; however, ocular administration is more efficient as it provides the mucosal immune responses with birds as mentioned above (Tong et al., 2001; Menendez et al., 2014; Thilakarathne et al., 2019; Beltrán et al., 2019; Gowthaman et al., 2020; Mo and Mo, 2025).

Vital vaccines utilize either viral vectored technologies (the fowlpox virus (FPV) and herpesvirus of turkeys (HVT) vectors) administered in ovo or subcutaneously at hatch, or live attenuated technologies when vaccine is chicken embryo origin (CEO) or embryonic tissue culture origin (TCO) (Mo and Mo, 2025). Although recombinant viral vector vaccines have become an important method of ILT prevention in many regions, particularly the United States (Tong et al., 2001), live attenuated vaccines remain a critical tool for global disease control. These vaccines are frequently applied in the form of eye-drops to induce robust local protection, often utilized as a booster for long-lived birds or during active field outbreaks (Thilakarathne et al., 2019; Gowthaman et al., 2020; Arachchige et al., 2022).

Live attenuated vaccines represent a formulation challenge because of the macromolecular complexity of viruses. Even though they have lost their virulence, they retain the ability to confer protective immunity against a virulent virus. To prevent unfolding, degradation, and aggregation of active substance, vaccines are formulated in an adequate cryoprotection medium of cryo/lyoprotectants, buffers, and other appropriate stabilizers, ensuring vaccine long-term stability (Hansen et al., 2015; Wang 2000). The lyophilized vaccine is reconstituted at time of need with a sterile liquid diluent consisting of suitable excipients, the vaccine is then poured into the diluent plastic bottle, fitted with a dropper, and administered ocularly. Generally, 30 mL of the reconstituted preparation contains a thousand drops representing a theoretical vaccine dose of 30 µL per bird.

The mass of eye drop produced from the conventional plastic dropper bottles fitted with the plastic dropper tips is influenced by three groups of factors (Van Santvliet and Ludwig, 2004): the design and characteristics of the dropper tip and bottle (Van Santvliet and Ludwig, 2001), the physicochemical properties of the solution to be dispensed (Van Santvliet and Ludwig, 1999b), and the handling of the dropper bottle (Van Santvliet and Ludwig, 1999b, Yoshikawa and Yamada, 2010).

In principle, drops are produced from eye dropper tips by the “constriction” of a liquid column. However, the common physical principles of drop formation should be considered as they can influence the final dose of preparation. In general, the weight of the drop (m) breaking away from the orifice of a vertical capillary and descending by the effect of gravity (g) is primarily a function of the surface tension of the liquid (σ) and a capillary radius (r) as pioneered by Tate (1864). At the instant of detachment, equilibrium exists between the gravitational force of a falling drop (*m·g*) and the capillary force (*σ∙2π∙r*) holding the drop at the end of the tube (Tate, 1864; Wang et al., 2020) as seen in Eq. 1.

(1) $$\begin{array}{c} m\cdot g=2\cdot \pi \cdot r\cdot \sigma \end{array}$$

This theoretical background, called Tate’s law, allows the prediction of drop mass if the surface tension of the liquid and a capillary radius are known (Šklubalová and Zatloukal, 2006). However, a small portion of the liquid remains attached to the tube due to the surface tension at the solid-liquid interphase, reducing the theoretical drop mass to the experimentally observed one when the drop is ejected (Wang et al., 2020). On the other hand, the occasional wetting of a dropper tip end can influence the drop size and, in consequence, the released dose of preparation due to the lateral drop formation. This phenomenon was observed especially for round dropper tips (Van Santvliet and Ludwig, 2001; Šklubalová and Zatloukal, 2006).

As no specific requirements regarding the drop size are required by Pharmacopoeias, the eye dropper tip designs and dimensions can vary significantly (Van Santvliet and Ludwig, 2004). Changes in the dimension of a dropper tip can alter the drop volumes markedly, whereas the inner diameter of the dropper tip orifice and the outer diameters represent the most significant parameters (Brown et al., 1985; Šklubalová and Zatloukal, 2006). The volume of solution instilled is, however, important as it directly influences the precorneal residence time and bioavailability of the vaccine/active substance as well as the risk of adverse effects in human and veterinary ocular pharmacotherapy (Davies, 2000; Vaajanen and Vapaatalo, 2017; Sebbag et al., 2019; Thilakarathne et al., 2019; Beltrán et al., 2019). In humans, the drop size varies within a large range of 30 – 60 μL (German et al., 1999); however, much smaller drops are expected for animals including birds (Sebbag et al., 2019).

Thousands of birds must be routinely vaccinated by careful administration onto the eye surface, avoiding an inefficient application when the drop rolls off the eye surface in larger-scale farming. To provide visual confirmation of successful delivery, a blue dye can be added to the vaccine diluent. Poultry farms rely on these resulting blue drops to ascertain the immunization of an acceptable quantity of birds within a given flock, which is crucial for achieving the desired vaccination rate (Korsa et al., 2018; Beltrán et al., 2019). The manual administration of eye drops by the operators requires converting a dropper bottle bottom up to produce a drop and squeeze it slowly by the fingers (Van Santvliet and Ludwig, 1999b). The vertical position of the bottle, i.e., dispensing angle 90°, is important to provide a uniform drop mass according to Tate's law (Wang et al., 2020). If the dispensing angle is decreased by the bottle tilting, the effective cross-sectional perimeter reduces leading to smaller drops (German et al., 1999; Van Santvliet and Ludwig, 2004; Šklubalová and Zatloukal, 2005).

It was demonstrated that the design of the dropper tip end can influence its lateral wetting wherever surface-active substances are used in the preparation. This results in the drop formation from the external surface of the tip and the production of paradoxically larger drops, which influences the administered dose (Šklubalová and Zatloukal, 2006). In large-scale vaccination, such an unexpected dose variability is challenging, and would finally be frustrating and exhausting for the operators. The study presented aims to compare a new apparatus that enables large-scale administration of doses for a larger flock model against conventional delivery techniques. Drop sizes and their variability were investigated for large-scale implementation of eyedropper medications dispensed from three manual conventional systems for ILT eye vaccines, namely, Nobilis® (P1), AviPro® (P2), and Poulvac® (P3), and compared with a new automated dropper device. The study focuses on the parameters of drop dispensing, such as the dispensing angle, the drop dispensing design simulating the dispensing rate, and the remaining volume, simulating the “life” of the product in a bottle. This experimental setup targets to mimic the conditions of drops administration with an emphasis on the variability, accuracy, and precision of drop formation, all having serious economic impact in achieving the adequate vaccination in commercial poultry industry.

## Materials and methods

### Materials

The vaccines analyzed include Nobilis® ILT (P1; MSD Animal Health, US), Avi-Pro® ILT (P2; a gift of Elanco US Inc., US), and Poulvac® ILT (P3; Zoetis UK Limited, United Kingdom), each lyophilized product, reconstituted with diluent provided by their respective manufacturers, Nobilis diluent for Nobilis and Avi-Pro, and Poulvac diluent for Poulvac. As this study utilizes of both types of live attenuated vaccines (CEO/TCO), the production method directly influences the size of the particles of active substance (infectious content) within each vaccine formulation. Before physicochemical measurements, therefore, the reconstituted vaccines underwent filtration through a 0.45 μm pore size membrane to eliminate all particles in the preparation. Measuring the drop mass under different designs (see below), the preparations were used in the complete composition (not filtered). All measurements were carried out following established protocols and using appropriate equipment.

### Density measurement

An oscillating U-tube density meter (DMA 4100 M, Anton Paar, Austria) was used to estimate density ρ (g/mL) at 25°C ± 0.1°C. By introducing 1 mL of the preparation sample into the oscillating U-shaped borosilicate glass tube of the equipment with a syringe, the density was measured five times for each preparation and the average, and standard deviation (SD) were computed. The SD for all samples was below 0.01 and, therefore, not included in the results presented.

### Surface tension measurement

A tensiometer, K100 (Krüss GmbH, Germany), was employed using the plate method at 25 °± 0.2°C; a clean Wilhelmy platinum plate was carefully placed in contact with the liquid level and submerged at a depth of 2 mm. From the force necessary to pull the plate out, the wetted plate length, and the contact angle between the plate and the liquid, the surface tension σ (mN/m) at the air/liquid interphase was estimated (SW LabDesk™, Krüss). The average of three repetitions (each with ten measurements) of the surface tension and SD were expressed.

### Viscosity measurement

An Ubbelohde capillary viscometer (Kavalier, Czech Republic) was used to determine the viscosity of all tested vaccine preparations. In agreement with European Pharmacopoeia 11.8 (2.2.9 Capillary viscometer method), the time for the defined volume of the liquid to pass through a capillary of a defined diameter area was measured at 25°C ± 0.5°C. An average of five subsequent measurements deviating within 2 seconds was accepted to estimate kinematic viscosity v (mm^2^·s^-1^) using Eq. 2.

(2) $$v=A\cdot t-\frac{B}{t}$$

where A is the viscometer constant (mm^2^∙s^⁻2^), t is the average flow time (s), and B/t represents the correction for kinetic energy (B = 2.8 was used according to the manufacturer's recommendation). The kinematic viscosity was conveniently converted into dynamic viscosity η (mPa·s) at the same experimental temperature by multiplying it by the liquid density ρ (g/mL).

(3) η=ν·ρ

The procedure was repeated five times for each preparation, obtaining both the average and SD.

### Experimental design

The manual drop dispensing using a conventional dosing system employs a plastic dropper bottle and a plastic tip. To evaluate the drop mass, three drop dispensing designs were used, hereafter called the *controlled rate dispensing* (CRD), the *fast rate dispensing* (FRD), and the *continuous pressure dispensing* (CPD), to investigate the effect of the dispensing rate. The combination with two other factors studied, the dispensing angle, DA, and the volume remaining in the bottle, RV, is shown in Table 1. Due to the factor combinations, 3^3^ = 27 experimental runs were implemented. In each experimental run, a minimum of 5 dropper tip replicates from the same design were used additionally to include the variability of the plastic device and account for possible defects in their structure. The CRD design, the upright position (DA = 90° from the horizontal), and a full bottle (30 mL) were considered the standard dispensing conditions for comparing the effect of all variables studied. For technical reasons, the experimental design was reduced for the automated dropper, as explained below.

**Table 1:** Description of experimental runs for influencing factors studied in dispensing three different vaccine medications with the conventional dosing systems.

Factor	Levels of Individual Factors		
Dispensing Rate Design	CRD (controlled rate dispensing)	FRD (fast rate dispensing)	CPD (continuous pressure dispensing)
Dispensing Angle	90°	65°	45°
Remaining Volume	30 mL (high)	15 mL (middle)	5 mL (low)

### Drop dispensing methods

The plastic dropper bottle was filled with the actual preparation volume (Remaining Volume in Table 1), fitted with a plastic dropper tip, and squeezed carefully in agreement with the dispensing design. In the CRD design, drops were produced under controlled slow manual compression with fingers placed in the middle of the dropper bottle to achieve a uniform drop production (Wang et al., 2020). The drop mass *m* (mg) of each drop (n = 10) was registered (analytical balance Entris 224I-1S, d = 0.1 mg, Sartorius Lab Instruments GmbH & Co. KG, Germany). The procedure was repeated ten times (n = 100). The average mass (n = 100) and SD were calculated. In the FRD design, the drops were produced by repeated quick manual compression using fingers in the middle of the dropper bottle until ten drops produced quickly were achieved. The drop mass *m* (mg) of ten collected drops was registered in ten separate runs; the average mass (n = 10) and SD were expressed. In the CPD design, the drops were produced as quickly as possible without the interruption of squeezing (i.e., with continuous compression of the bottle). The drop mass *m* (mg) of ten collected drops were registered; the average mass (n = 10), and SD were calculated.

**Fig. 1:**
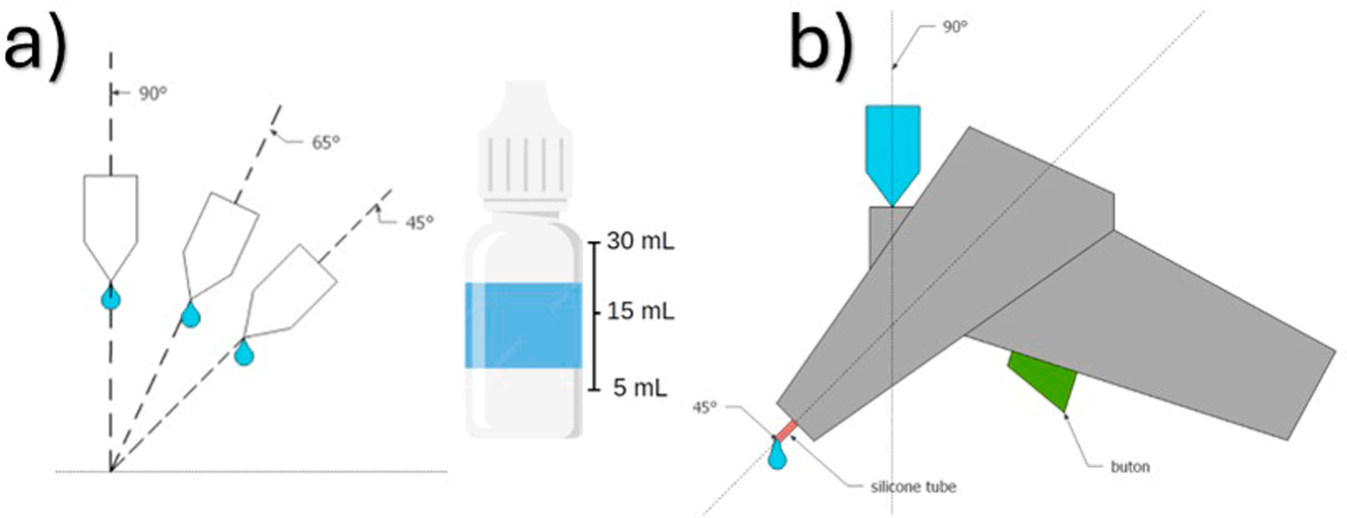
Schematic illustration of drop dispensing using the conventional method at different dispensing angles (DA) and remaining volumes (RV) in A) and using the automated dropper automated dropper in B). Details are explained in the text.

Eye drops were produced using the three dispensing rate designs described above under three DAs: at an upright bottle position (90°, bottom-up), tilting from vertical position to 65° and 45°, respectively (Fig. 1A). The influence of the remaining volume, RV (mL), was evaluated so that the dropper bottle was filled with 30 mL of the preparation (high volume), 15 mL (middle volume), and 5 mL (low volume), respectively, with each of the dispensing rate designs and DAs tested. The volume of preparation in the bottle was maintained by replacing the preparation after two experimental runs.

### Automated dispensing method

A new automated dropper device (AviPro® Eye Dropper, an automated dropper device, Henke-Sass, Wolf GmbH, Germany) for the eye drop’s production was used to compare it to a manual administration. The apparatus was fully charged prior to experimental runs. The dropper bottle filled with the actual volume of the preparation was attached by a collar to the equipment and located in the required upright position (bottom-up) (Fig. 1B, Fig. 4j). Following the user manual instruction, the apparatus nozzle was deaerated. This procedure was repeated whenever the preparation volume was changed/refilled with the actual preparation volume (Table 1). A silicone tube was attached to the equipment instead of a dropper tip to produce a drop and the air from the nozzle was released by pressing a trigger button. The apparatus was calibrated before measurement by measuring pipette to express the number of drops per milliliter.

As the position of the bottle in the automated dropper requires keeping the bottle in the bottom-up position (Fig. 1B, Fig. 4j), the influence of the DA cannot be studied; the dropper silicone tube is always at 45° from horizontal. As the apparatus produces drops by pressing a button, the *controlled rate dispensing apparatus* (CRDA) design shall indicate that ten individual drops were produced by pressing the trigger button. The drop mass *m* (mg) of each drop was registered (analytical balance, n = 10), the procedure was repeated ten times (n = 100), and the average and SD were calculated. This procedure simulates the real administration of vaccine drop with one operator administering the vaccine and the other assisting with bird handling. In the *fast rate dispensing apparatus* (FRDA) design, ten consecutive drops were produced by quick pressing the trigger button with at least 1 second in between, and the total mass of the ten collected drops were measured. The 1 second interval used in the FRDA design was selected to serve as an extreme model of high-speed application. Because real-world vaccination rates are physically limited by the time required for an assistant to catch, position, and release each individual bird, this continuous 1 second testing interval serves to validate the device's dosing consistency at a speed that exceeds the maximum practical limits of field application. This procedure was repeated ten times, and the average mass (n = 10), and SD were computed. However, the *continuous pressure rate dispensing* design could not be used and evaluated.

### Statistical analysis

The results were statistically analyzed using MS Excel and SciPy Python scripts. For Analysis of Variance (ANOVA), the data first underwent a normality check via the Shapiro-Wilk test; non-normally distributed variables were then transformed using methods like log or square root transformations before ANOVA was performed to identify statistically significant differences in mean drop mass, dose variability, and dispensing rate across various eye drop preparations and administration methods, all at a 0.05 significance level. Furthermore, multilinear and non-linear regression analysis with interaction terms, utilizing *statsmodels* in Python, was employed to explore and quantify the relationships between key variables such as dispensing angle, dropper design, and their influence on drop mass and its variability (i.e., variability of dose). To compare the various models properly, an adjusted R^2^ value was used to account for multiple factors.

## Results and discussion

Eye drops are commonly used for the treatment of various eye conditions in animals. They are also increasingly being utilized for the vaccination of poultry providing the first line of defense against viral infections, such as ILT (Tong et al., 2001; Devlin et al., 2008; Menendez et al., 2014; Thilakarathne et al., 2019; Beltrán et al., 2019; Tong et al., 2020; Gowthaman et al., 2020; Mo and Mo, 2025. Producing eye drops precisely and consistently is crucial in ensuring an effective vaccination process. This study introduces new automated dropper equipment for the larger-scale eye drop administration. As many factors influence the drop sizes dispensed from the manual conventional systems and their variability, the parameters of drop dispensing are investigated with conventional administration systems for three commercial ILT eye vaccines, P1 – P3. The achieved drop masses and dose variability are compared with those obtained by an automated dropper equipment.

### Physicochemical properties

Ophthalmic preparations generally contain the necessary excipients to modify their properties regarding physiological acceptability (e.g., tonicity and pH value) and to avoid any deleterious effect on the cornea (Kompella et al., 2010). However, active substance stability must also be considered, which is particularly important during freeze-drying of live viruses (Hansen et al., 2015; Wang, 2000). These additives influence the physicochemical properties of eye preparation, such as density, viscosity, and surface tension that may affect the mass of the eye drop and the uniformity/variability of its production (Van Santvliet and Ludwig, 1999b). Before evaluating the effectiveness of the delivery systems, it is therefore necessary to investigate the physicochemical properties of the preparations.

This study utilizes both types of live attenuated vaccines (CEO/TCO), which were freeze-dried products. The production method directly influences the size of the particles of active substance (infectious content). Particularly, CEO type vaccines, made from tissue homogenates, can contain macroscopic particles (Gowthaman et al., 2020; Mo and Mo, 2025). To ensure the accuracy and precision of the analytical methods during testing, the vaccines were reconstituted with their specific diluents and then filtered to remove these larger particles before measurement. The results are summarized in Table 2. A direct comparison between filtered and unfiltered preparations was not performed as vaccine particles would interfere with the measurements.

**Table 2:** The physicochemical properties of the preparations (SD in brackets); dropper tip orifice diameter (r) is included.

Preparation	Density ρ (g/mL)	Viscosity		Surface Tension, σ (mN/m)	Orifice diameter, r (mm)
		ν (mm^2^/s)	η (mPa·s)		
P1	1.0075	0.971 (0.010)	0.975 (0.011)	38.134 (0.263)	1.4
P2	1.0046	1.157 (0.003)	1.173 (0.003)	48.365 (0.175)	1.5
P3	1.0166	0.987 (0.003)	0.989 (0.003)	40.301 (0.074)	0.8

Eye drops generally are composed of an aqueous solution with a water-like density. This was also observed for the ILT vaccine preparations; the highest value of 1.0166 g/mL was noted for P3, still only slightly above water density at identical temperature. Viscosity modifiers (polymers) can be used in ophthalmic drops to prolong the contact time with the cornea (Doughty and Glavin, 2009; Kompella et al., 2010; Nguyen et al., 2022). In the lyophilized products, polymers are also often used to protect the active substance from the adverse effects of frost and drying during the lyophilisation procedure (Hansen et al., 2015; Wang 2000). The results observed by the measurement of kinematic viscosity and calculation of the dynamic one (Eq. 3) are very slightly below or above water viscosity at similar conditions (1 mPa·s). The lowest dynamic viscosity was observed for P1, while the highest value of 1.173 mPa·s was detected for P2 (Table 2). As the influence of viscosity on the drop mass has been referred to as significant if the viscosity of the preparation is above approximately 25 mPa·s (Van Santvliet and Ludwig, 1999a), no effect of viscosity is expected for the evaluated vaccine preparations.

Based on the theory of Tate’s law, the effect of surface tension on the drop mass is crucial. The lower surface tension, the smaller drops are obtained. Surprisingly, Šklubalová and Zatloukal (2005) detected the effect of surface tension to be non-significant in a systematic factorial designed experiment. This deviation from the general situation was due to the used experiment with two levels only where the more significant effects of the dispensing angle and the dispensing rate overpower the effect of surface tension. As seen in Table 2, the following trend in surface tension was observed: γ(P2) > γ(P3) > γ(P1). Eye drops may contain excipients to adjust the properties related to physiological acceptability to the eye and to stabilise the preparation (Pogorzelski et al., 2012). All these additives may generally affect the physico-chemical properties. Being a member of the Herpesviridae family, an enveloped ILT virus (Beltrán et al., 2019; Mo and Mo, 2025) can be easily inactivated by surface-active substances. In lyophilisate ILT vaccine products, they must therefore be used with caution. Based on the assumption that the vaccine is a single-use preparation, when all doses are administered within the same time period after dilution, the preparation does not contain the preservative. By contrast, some peptides and/or amino acids attributable to residual host cell proteins and lipids from the culture process or used in freeze-drying to form an acceptable cake, may be sources of surface activity (Hansen et al., 2015; Patel et al., 2017; Wang, 2000). The blue dye lacks surfactant properties and is included strictly as a visual marker.

**Fig. 2:**
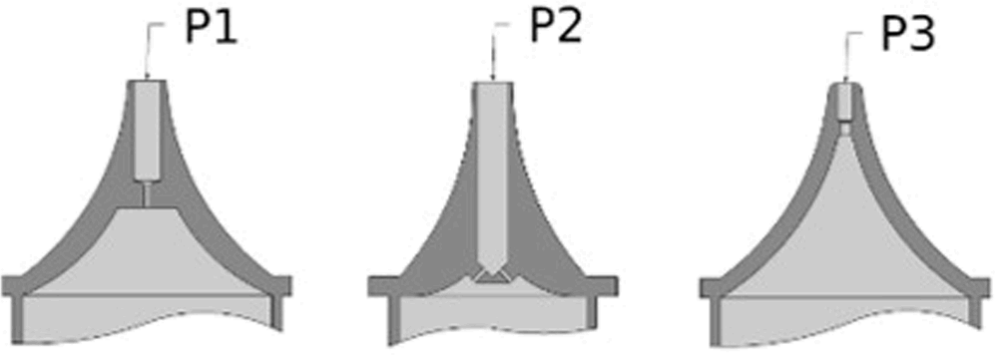
Graphical representations of the internal and external features for the three investigated dropper tips: P1 (left), P2 (center), and P3 (right).

The characteristics of the dropper tip, particularly the outer diameter of the orifice, as well as its design influence the drop mass significantly (Van Santvliet and Ludwig, 2004; Šklubalová and Zatloukal, 2005; Šklubalová and Zatloukal, 2006). For this reason, Table 2 includes the diameters (r) of the dropper tips used specifically for each preparation. It is more prominent for P1 and P2 but smaller for P3. At the vertical position of a dropper bottle (bottom up, DA 90°), a drop is formed at the perimeter of a dropper tip with a diameter equal to r. As many designs for dropper tips have been described over the years, the shape of the dropper tip end must also be considered in predicting the drop mass (German et al., 1999; Van Santvliet and Ludwig, 2001). The flat end influences the drop mass whenever the wetted surface increases the perimeter from the inner tip diameter to the outer one (German et al., 1999; Šklubalová and Zatloukal, 2005). On the other hand, the round-shaped tip end might increase the drop mass by the lateral surface drop formation wherever the drop is produced under tilting, i.e., if decreasing the DA from vertical position (Van Santvliet and Ludwig, 2004; Šklubalová and Zatloukal, 2005) This becomes relevant particularly if the preparation surface tension is low, e.g., in the presence of surfactants. As illustrated in Fig. 2, flat tips were used for P1 and P2, while a round one was used for P3.

The occasional, unexpected wetting of the dropper tip end surface contributes to the drop size increase and the higher dose variability (Šklubalová and Zatloukal, 2005; Šklubalová and Zatloukal, 2006). However, there are safety concerns for using a flat versus round tip design as the flat tip design has more of a potential for injury risk, especially when the eye is concerned (Van Santvliet and Ludwig, 2001). Apart from the physicochemical properties of the dispensed preparation and the design and dimensions of the dropper tip, which are carefully chosen by the manufacturer, the actual eye drop mass is affected strongly by the user’s manipulation technique (Van Santvliet and Ludwig, 1999b; Van Santvliet and Ludwig, 2004). To form a drop, generally the bottle should be inverted and squeezed carefully until a drop descends. Under such circumstances, the slow drop formation follows Tate´s law and the drop mass can be controlled (Wang et al., 2020). This is the case for CRD design, and it should be noted that CRD was shortly trained before experimentation to avoid an occasional change in the drop formation rate. For the investigation, the drop mass was selected instead of drop volume, as mass is independent of temperature and can be easily measured by the balance.

**Fig. 3:**
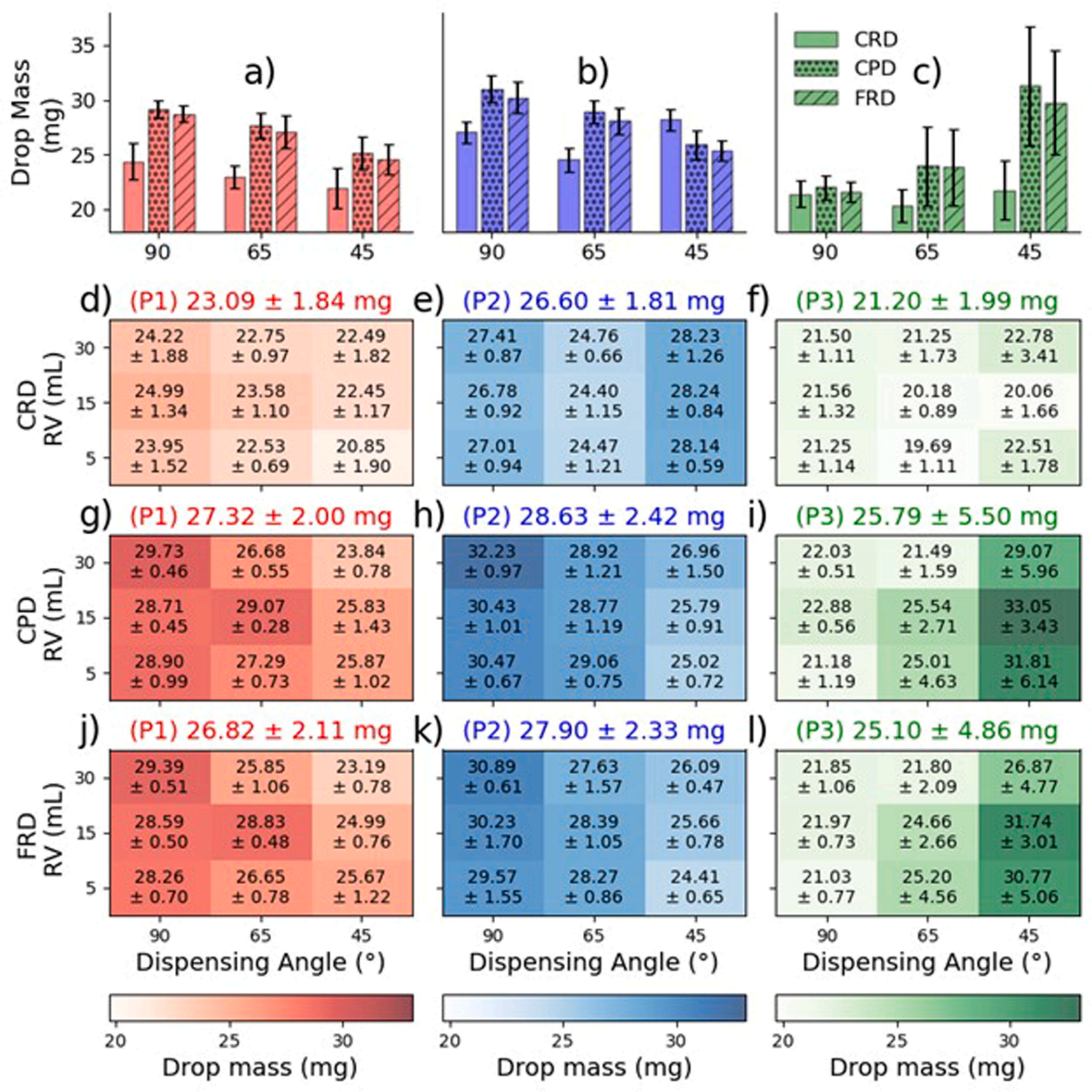
Analysis of drop mass for three preparations (P1-P3) across various dispensing designs. (Top, a-c) Bar charts displaying the drop mass means and standard deviations for all remaining volumes at different dispensing angles for the controlled-rate dispensing (CRD, a), fast-rate dispensing (FRD, b), and continuous-pressure dispensing (CPD, c) designs. (Bottom, d-l) Drop mass means observed including remaining volume (RV) as a separate influence. Results in the heat map displayed as average ± standard deviation.

While in the previous section we focused on the influence of the given preparation on drop mass, here we extend the investigation with the additional factors dispensing rate, angle and remaining volume. The results of our experiments with these factors are summarized in Fig. 3.

### Influence of preparation

Based on the average drop masses by preparation for CRD (Fig. 3d-f), P2 yielded the highest drop mass *m*(P2) (26.60 ± 1.81 mg), followed by *m*(P1) (23.09 ± 1.84 mg), and the lowest drop mass *m*(P3) was obtained from P3 (21.20 ± 1.99 mg). This trend*, m*(P2) > *m*(P1) > *m*(P3) was consistent, regardless of dispensing rate utilized. For CPD and FRD, see (Fig. 3g-l). This agrees with the theoretical expectations for the highest surface tension of preparation and the largest orifice diameter in P2 (Table 2), and the smallest being P1.

### Comparison with Tate’s law predictions

The same trend was predicted based on Tate’s Law (Eq. (1)), whose application yielded *m*(P2) = 46.5 mg > *m*(P1) = 34.2 mg > *m*(P3) = 20.6 mg. However, there is significant discrepancy of these predictions with experimental observations for the flat-tipped droppers (P1 and P2). The difference between the calculated mass and the experimentally recorded one is a function of the orifice diameter which is larger for the flat-tipped droppers (1.4 and 1.5 mm for P1 and P2, respectively), and the liquid surface tension (Table 2) (Wang et al., 2020).

### Influence of dispensing rate

The handling of the dropper bottle by a user is an essential factor influencing the mass of the drop and hence the efficiency of the vaccination or therapy (Van Santvliet and Ludwig, 1999b; Van Santvliet and Ludwig, 2004; Šklubalová and Zatloukal, 2005; Yoshikawa and Yamada, 2010; Thilakarathne et al., 2019; Gowthaman et al., 2020). Contrary to the spontaneous flow of a liquid from the capillary tube by the influence of gravity, eye dropper systems should produce the drops only when the bottle is compressed. This force applied to the bottle wall influences the rate of drop formation (Van Santvliet and Ludwig, 2001). It has been demonstrated that the greater force leads to a faster dispensing rate followed by an additional pulse of a liquid increasing the drop mass (Jho and Burke, 1983; Jho and Carreras, 1984; Wang et al., 2020). Regardless of preparation, the trend for dispensing rate was *m*(CPD) > *m*(FRD) > *m*(CRD).

### Influence of dispensing angle

**Fig. 4:**
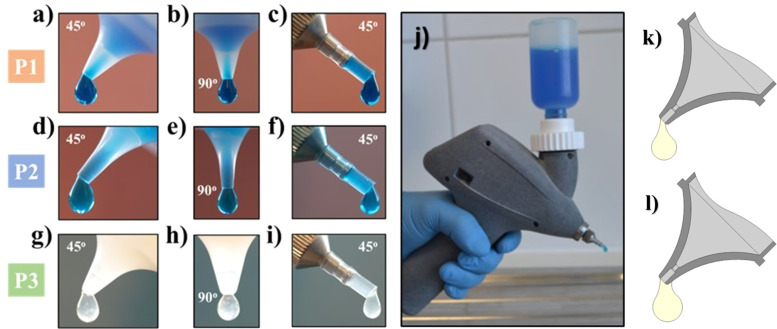
Visualization of the vaccine delivery from dropper tips in the preparation P1 (a-c), P2 (d-f) and P3 (g-i) at different dispensing angles using a manual (a, b, d, e, g, h) or automated device (c, f, i) delivery. Illustration of the droplet's path on the external surface for the round tip: (l) "unwetted" tip, (k) "wetted" tip with the lateral surface formation. The AviPro® Eye Dropper automated device utilized in the P2 vaccine delivery.

The dispensing angle showed a different trend when comparing the flat-tipped droppers (P1/P2) and the round-tipped dropper (P3). The former showed a positive relationship between the angle and drop mass, while the latter (P3) showed a negative relationship (Fig. 3a-c). When the bottle is tilted, the efficient horizontal perimeter decreases leading to the smaller drop for flat-tipped capillary (Fig. 4a, d). For round-tipped dropper, a drop can form similarly if the dropper tip surface is not wetted (Fig. 4k, “non-wetted”). Due to the lateral wetting, the drop slides along the wetted outer surface (Fig. 4l, "wetted") and spreads along the round-tip leading to drop detachment from the external surface of the tip. This resulted in the production of the paradoxically larger drops at more tilted angles in case of P3 (Fig. 4g). This specific influence has been observed in other investigations for such droppers (Šklubalová and Zatloukal, 2006) and should be a consideration when choosing application systems for eye drop formulation and administration, particularly whenever the surface tension of the preparation is low.

### Influence of remaining volume

No noticeable pattern for the influence of the variable remaining volume on drop mass was directly observable from Fig. 3.

While the mean drop mass provides a general measure of dose, its variability is of greater importance to the commercial poultry industry. For manufacturers, reliability of the dropper is the key concern. Companies can adjust vaccine formulations or administration protocols to account for differences in average drop mass between preparations. However, inconsistent dosing can lead to some birds being under-vaccinated, leaving them susceptible to diseases like Infectious Laryngotracheitis (ILT), while others are over-vaccinated, resulting in unnecessary vaccine waste. Therefore, understanding and controlling the factors that cause dose variability is critical for ensuring the efficacy of large-scale vaccination programs. It was difficult to make any relevant and consistent conclusions based on the observed standard deviations expressed in Fig. 3. The only notable exception (Fig. 3c) was in P3, which can be attributed to the lateral-wetting previously discussed, which can be observed visually in Fig. 4l.

### Candidate regression models

Regression analyses were implemented to provide a more comprehensive explanation for the data variance, which is not easily discernible from Fig. 3. When fitting regression models to the pooled data of the entire set of observations (Fig. S1), comparison of models revealed that the variables preparation (P in Fig. S1) and dispensing rate (R in Fig. S1) contributed most significantly to the observed drop mass variance. When we separate the data into all distinct combinations of dispensing rate and preparation and fit individually to the corresponding subsets of data, we obtain additional models, the adjusted r^2^ of which can be seen in Fig. S2. Each of the models in Fig. S2 show a different amount of explained variance and for models for CRD with preparations P2/P3, clearly no model is able to capture the variance in the drop masses. For CRD, only P1 shows a noticeable fit, though with a poor adjusted r^2^ value of less than 0.3. However, only when the manual dropper is utilized with a non-recommended dispensing rate (namely either CPD or FRD) does any significant amount of the variability begin to be explained with an adjusted r^2^ of up to 0.784. The change in the amount of variability which can be explained by these models further justifies the implementation of the droppers at the *recommended* dispensing rate, as the risk for systemic delivery error drastically increases when dispensing angle or remaining volume have a larger influence on drop mass variability.

### Regression analysis with selected models

**Fig. 5:**
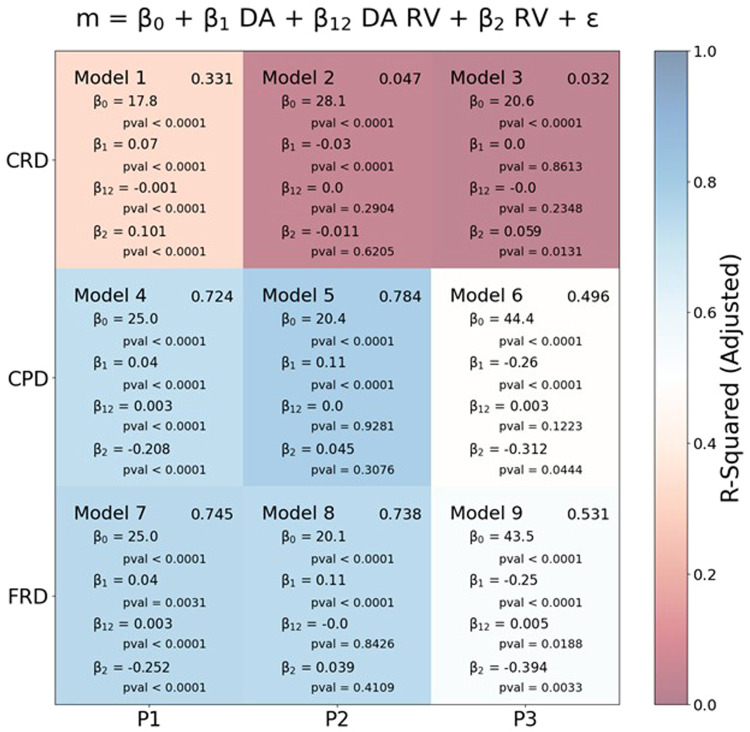
Coefficients and p-values obtained from models using the equation $m\approx \beta_{0}+\beta_{1}\text{DA}+\beta_{12}\text{DA}\;\text{RV}+\beta_{2}\;\text{RV}+\varepsilon$. Each model was fit individually to subsets of data separated by all distinct combination of preparation (P1-P3) and dispensing rate (CRD/FRD/CPD).

**Fig. 6:**
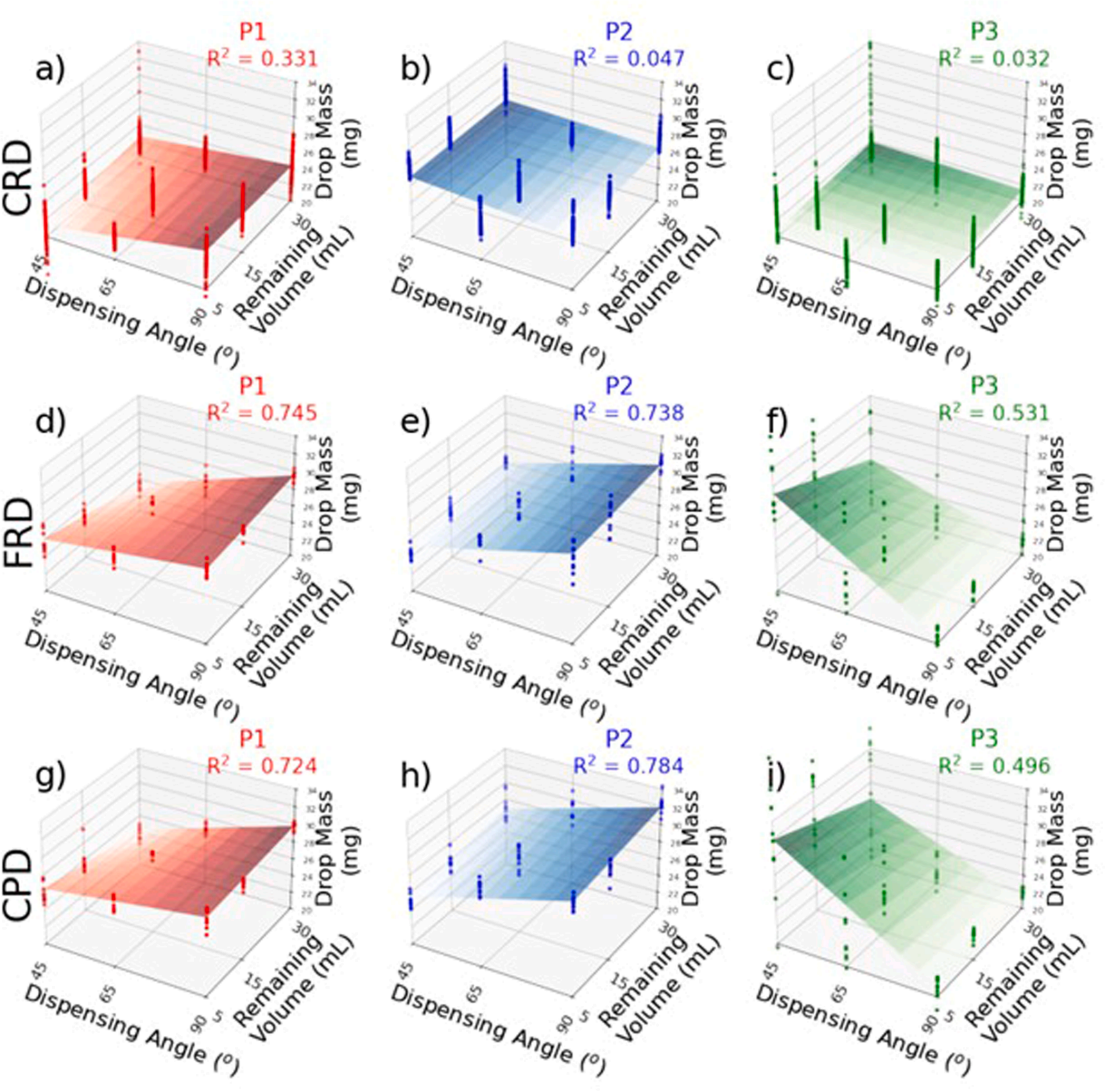
Surface plot graphs of the relationship between the drop mass (m) and the dispensing angle (A) and remaining volume (V) using the equation, $m\approx \beta_{0}+\beta_{1}\text{DA}+\beta_{12}\text{DA}\;\text{RV}+\beta_{2}\text{RV}+\varepsilon$, for both the manufacturer recommended controlled-rate delivery (CRD) in a-c, as well as the fast-rate delivery (FRD) in d-f and the continuous-pressure delivery (CPD) in g-h. Also included are the amount of explained variance by the models in adjusted R^2^.

Out of the models tested in Fig. S2, the equation $m\approx \beta_{0}+\beta_{1}\text{DA}+\beta_{12}\text{DA}\;\text{RV}+\beta_{2}\;\text{RV}+\varepsilon$ was used for the following analysis, compiled in Fig. 5, where *m* represents the drop mass (mg), DA represents the angle, RV represents the remaining volume, and ε represents unexplained variance. Including the interaction term between angle and volume allows the model to capture a more complex non-linear relationship than just the sum of the individual effects of angle and volume. By expressing the data in such a way, we were able to quantify the trends previously described (CRD variance is poorly explained by any model, and that the influence of angle on drop mass is inverse between the flat and round-tipped droppers) as well as an interesting influence of remaining volume that only occurs for P1 at non-recommended dispensing rates (FRD/CPD). No significant effect of the remaining preparation volume on the drop size was expected when a drop from conventional eye drops’ flexible plastic dropper systems is formed by squeezing (Šklubalová and Zatloukal, 2006), so this was a relatively unexpected finding from the regression analysis. Each of the models described in Fig. 5 can be seen plotted as surface response plots in Fig. 6.

### Influence of bottle stiffness

It should be noted that the stiffness of the plastic bottle is important for the users and their compliance in the preparation dispensing. However, any veterinarian or poultry technician producing a drop manually from the plastic dispensing bottle appreciate if the bottle is acceptably easy to squeeze. From this point of view, the P1 bottle material had a disadvantage when scaling up the number of drops. Based on our qualitative observations, the plastic bottle was stiff and had limited flexibility, which made the squeezing tough. In this case, it might also be difficult to control the drop dispensing rate due to fatigue, which could increase the dose variability, particularly if many drops must be produced within a limited time, such as in the pullet vaccination.

**Fig. 7:**
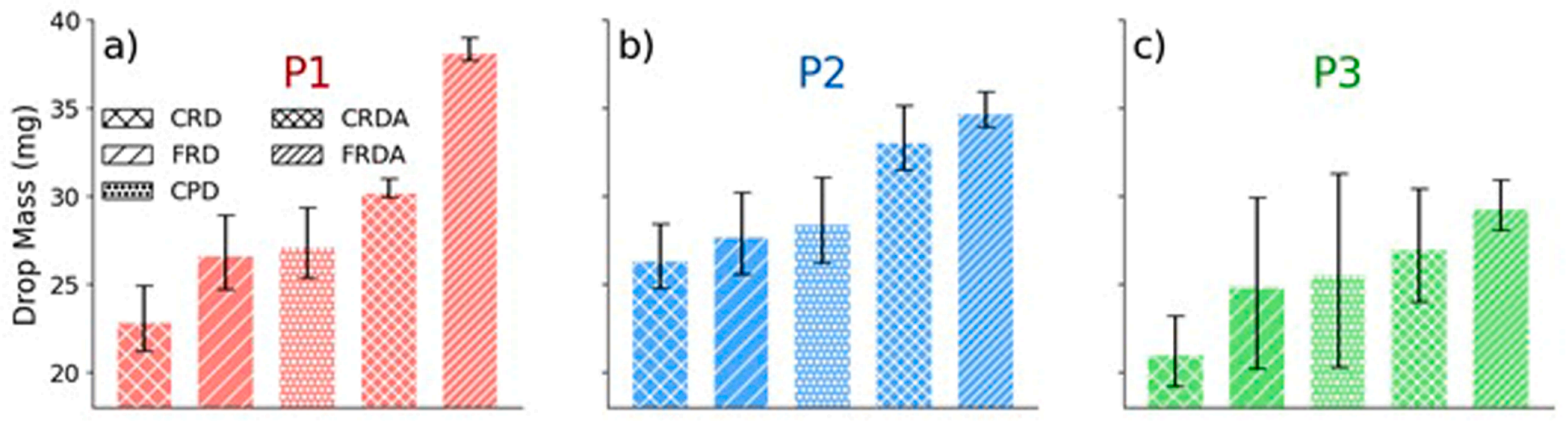
Bar charts represent the average and standard deviation drop mass for each of the preparations (P1-P3), using both the conventional dropper with multiple dispensing rates (CRD, FRD, CPD), and the automated device (CRDA, FRDA).

As tens of thousands of birds might be vaccinated routinely simultaneously in the poultry industry and the manual administration of eye drops is demanding for operators, a new automated device is introduced and evaluated in this work. Like with manual administration, the factors influencing drop mass were investigated for the vaccine preparations P1, P2 and P3. Due to the restrictive nature of the automated dispensing device (Fig. 1B, Fig. 4j), the drop is produced from the silicone capillary at the dispensing angle of 45^o^. Nevertheless, the average drop mass was compared with that achieved at the vertical position (90°) for the manual systems, because such is the standard recommended DA for these systems. Although the effective horizontal perimeter decreases at a 45° dispensing angle, resulting in a smaller droplet for a flat-tip capillary, such as the silicone tube of the automated device, larger drops were detected because the diameter of the silicone tube is larger (3.0 mm) (Fig. 7).

**Fig. 8:**
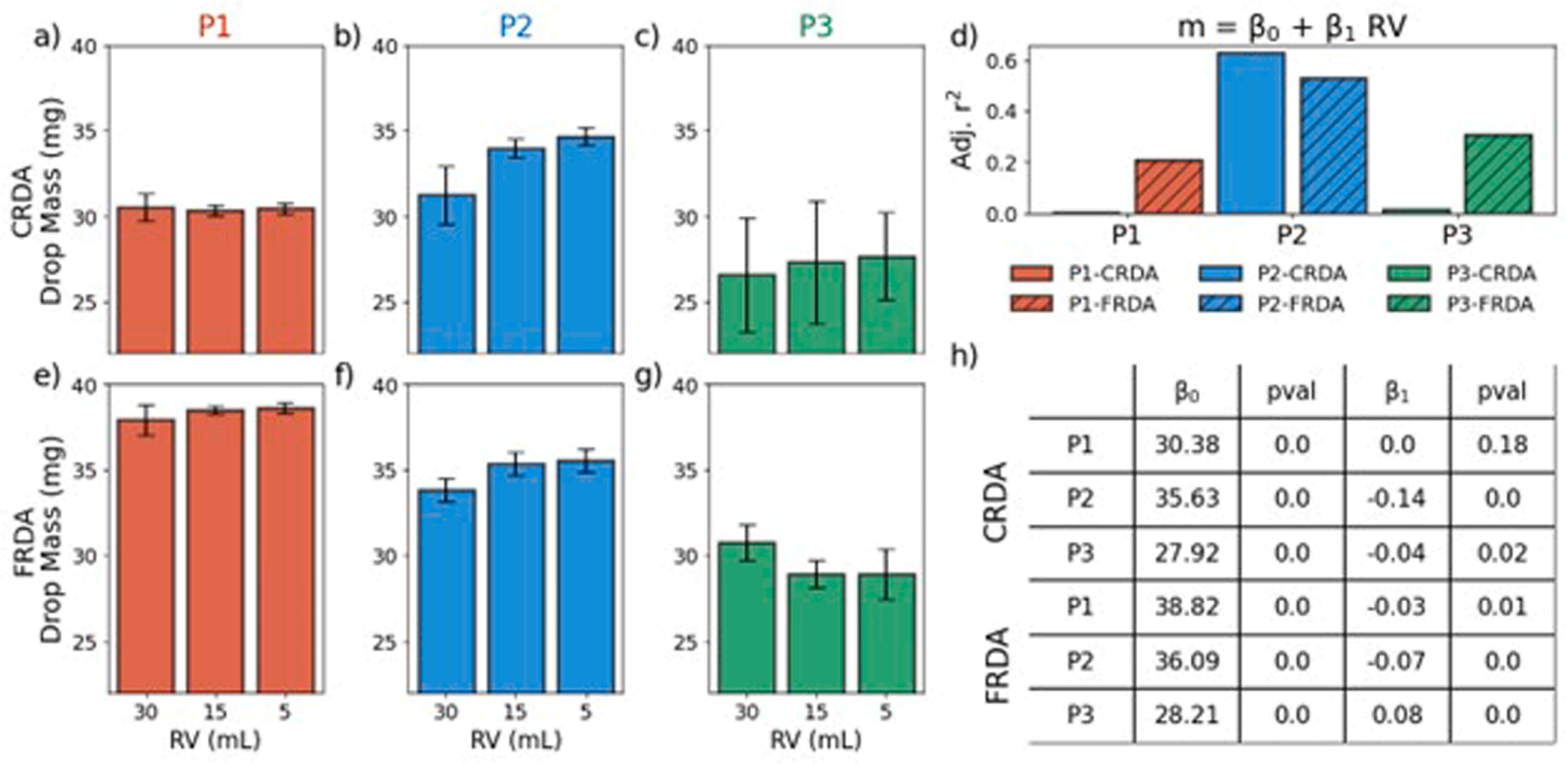
Bar charts of the average mass obtained using the CRDA (a-c) and FRDA (e-g) for each preparation, as well as the respective fits (adjusted r^2^) from models using datasets separated for each distinct combination of preparation (P1-P3) and dispensing rate (CRDA/FRDA) fit independently (d) to the equation, $m=\beta_{0}+\beta_{1}\text{RV}$. Also included are the coefficients and p-values obtained from each model (h).

The results from this investigation of the automated dropper (Fig. 8) showed that there is very little influence of the remaining volume when either P1 or P3 are used with the manufacturer recommended delivery technique (CRDA). This is evidenced by the bar chart in Fig. 8a, the low r^2^ value of the fit of the model to the equation $m=\beta_{0}+\beta_{1}\text{RV}$ (Fig. 8d). This is particularly true for P1, as it gives the only p-value that indicates it is statistically insignificant (0.18). While the results do show that the automated dropper improves precision and decreases variance factors for the manufacturer recommended preparation and delivery technique, the models also show a relationship that forms between the remaining volume and drop mass when P2 is used. It should be advised that the ideal use of the automated dropper device is using CRDA with P1 to achieve best results.

There were also several advantages observed personally using the automated dropper. The prescriptive nature of the device removes the consideration of angle as a possible source of variance in the system. Additionally, the easier grip of the device allows for rapid and easily reproducible drop masses from each use of the device. This is further compacted by the standard deviations obtained from CRDA and FRDA, which were both substantially lower than from manual delivery, particularly when compared to alternative delivery techniques such as FRD or CPD (Fig. 7). Due to the flat-tipped nature of the device, much of the problematic wetting observed by P3 was no longer an issue, which is an additional advantage of the automated device. Despite multiple advantages of the automated dispenser, the correct utilization of the dispenser is still of concern. Depending on whether the apparatus is used in a controlled (CRDA) or rapid model (FRDA) manner, the average drop masses can vary depending on the preparation used; from 30.45 mg (CRDA) to 38.34 mg (FRDA) with P1, to as little as 33.30 mg (CRDA) to 34.90 mg (FRDA) in P2. This means that despite removing remaining volume or dispensing angle as major sources of error, considerations must still be paid towards the dispensing technique, depending on the physico-chemical properties of the preparation.

In conclusion, this study addressed the critical challenge of ensuring accurate and decreasing the variance for ocular vaccine delivery in large-scale poultry farming, a process currently hampered by the inherent user-dependent variability of manual eye-drop administration. We employed a robust experimental design to evaluate the factors influencing drop mass and variability across three manual vaccine preparations and delivery systems: Nobilis (P1), AviPro (P2), and Poulvac (P3). Our findings demonstrate the potential of automated delivery technology to significantly enhance the accuracy and precision of mass vaccination programs, thereby reducing waste, lowering labor costs, and improving animal health outcomes. However, some vaccine loss occurs due to the necessary deaeration procedure and calibration of the device when a bottle with preparation (30 mL) is attached. Given that each bottle will process thousands of doses, this is a negligible amount.

In manual dispensing, the dispensing angle and the dispensing rate of production emerged as the primary sources of drop size variability. This dose variance was further compounded by the dropper tip design (flat vs. round). For the flat-tipped droppers (P1 and P2), decreasing the dispensing angle led to smaller drops, while the round-tipped P3 exhibited an unexpected increase in drop mass and variability at tilted angles (65^o^/45^o^) due to problematic lateral wetting. Furthermore, manual dispensing techniques not recommended by the manufacturer such as Fast-Rate and Continuous-Pressure Designs (FRD/CPD) drastically increased mass variability across all preparations. Statistical modeling confirmed that the dispensing angle and rate were the most significant drivers of drop mass variability in manual delivery. In contrast, the remaining volume of the vaccine in the bottle was consistently found to have a negligible influence on drop mass or variability across all systems.

Based on our personal experience, the automated dropper apparatus demonstrated superior performance, achieving lower standard deviations in drop mass compared to any manual delivery method, particularly when contrasted with alternative manual techniques (FRD/CPD). This improved consistency stems from the apparatus’ prescriptive nature, which eliminates the dispensing angle and dispensing rate as sources of variance, facilitates reproducible drops through an easier grip, and mitigates lateral wetting issues through its flat-tipped design. Although the drops were larger due to the automated device’s wider silicone tube (3.0 mm versus 0.8-1.5 mm), the implementation of the automated device resulted in a statistically significant reduction in drop mass variance which may offer a practical advantage for large-scale applications.

Since many eye-drop vaccines are still applied manually, the recommended handling for manual users to reduce the variability in dropper size is as follows: The bottle should be converted to a vertical position (bottom up) and held in this position during application of the drops. Hold the bird so that one eye is pointing upwards. To produce a drop, the bottle should be carefully squeezed in the middle of the bottle body until one drop of vaccine falls into the eye.

## CRediT authorship contribution statement

**Michael Bakker:** Writing – review & editing, Writing – original draft, Visualization, Formal analysis, Data curation. **Barbora Vraníková:** Writing – review & editing, Supervision, Project administration, Methodology, Conceptualization. **Erik Jurjen Duintjer Tebbens:** Writing – review & editing, Formal analysis, Data curation. **Zdenka Šklubalová:** Writing – review & editing, Visualization, Validation, Supervision, Project administration, Methodology, Investigation, Funding acquisition, Conceptualization.

## Disclosures

The authors declare that they have no known competing financial interests or personal relationships that could have appeared to influence the work reported in this paper.

## Appendix Supplementary materials

Image, application 1

## Footnotes

Supplementary material associated with this article can be found, in the online version, at doi:10.1016/j.psj.2026.107159.
