# Supplementary material for: Comparative analysis of drop mass variance for manual and automatic delivery of large–scale eye drop poultry vaccine
Source: Poult Sci. 2026 May 21;105(11):107159. doi: 10.1016/j.psj.2026.107159 (PMC13449668; doi:10.1016/j.psj.2026.107159)
Supplement: Supplementary file 1 [file mmc1.docx]

# SUPPORTING INFORMATION

The following section contains the Supporting Information referenced in the main article.

**Comparison of Multilinear Regression Models.**

**Figure S1.** Comparison of multi-linear regression models where the independent factors are various combinations of preparation (P), rate (R), dispensing angle (DA), and remaining volume (RV) and drop mass is the dependent variable. The ability to explain variability is expressed by their adjusted R^2^ values and BIC values are displayed for further comparison.

**Figure S2.** Comparison of multi-linear regression models where the dataset is separated into all distinct combinations of (P1-P3; top to bottom) and dispensing rates (CRD, FRD, and CPD; left to right) and fit individually to the corresponding subsets of data using various combinations of dispensing angle (DA), and remaining volume (RV) as variables expressed as their respective fit (adjusted R^2^) based on mass. Interactions between variables are noted (A:B) with colons.
